# HDAC inhibitors Panobinostat and Romidepsin enhance *tax* transcription in HTLV-1-infected cell lines and freshly isolated patients’ T-cells

**DOI:** 10.3389/fimmu.2022.978800

**Published:** 2022-08-16

**Authors:** Annika P. Schnell, Stephan Kohrt, Aris Aristodemou, Graham P. Taylor, Charles R. M. Bangham, Andrea K. Thoma-Kress

**Affiliations:** ^1^ Institute of Clinical and Molecular Virology, Universitätsklinikum Erlangen, Friedrich-Alexander-Universität Erlangen-Nürnberg, Erlangen, Germany; ^2^ Section of Immunology of Infection, Department of Infectious Disease, Imperial College London, London, United Kingdom; ^3^ Section of Virology, Department of Infectious Disease, Imperial College London, London, United Kingdom

**Keywords:** HTLV, *tax*, latency, latency-reversing agent, HDAC-inhibitor, HAM/TSP, Panobinostat, Romidepsin

## Abstract

The viral transactivator Tax plays a key role in HTLV-1 reactivation and *de novo* infection. Previous approaches focused on the histone deacetylase inhibitor (HDACi) Valproate as a latency-reversing agent to boost Tax expression and expose infected cells to the host’s immune response. However, following treatment with Valproate proviral load decreases in patients with HAM/TSP were only transient. Here, we hypothesize that other compounds, including more potent and selective HDACi, might prove superior to Valproate in manipulating Tax expression. Thus, a panel of HDACi (Vorinostat/SAHA/Zolinza, Panobinostat/LBH589/Farydak, Belinostat/PXD101/Beleodaq, Valproate, Entinostat/MS-275, Romidepsin/FK228/Istodax, and MC1568) was selected and tested for toxicity and potency in enhancing Tax expression. The impact of the compounds was evaluated in different model systems, including transiently transfected T-cells, chronically HTLV-1-infected T-cell lines, and freshly isolated PBMCs from HTLV-1 carriers *ex vivo*. We identified the pan-HDACi Panobinostat and class I HDACi Romidepsin as particularly potent agents at raising Tax expression. qRT-PCR analysis revealed that these inhibitors considerably boost *tax* and Tax-target gene transcription. However, despite this significant increase in *tax* transcription and histone acetylation, protein levels of Tax were only moderately enhanced. In conclusion, these data demonstrate the ability of Panobinostat and Romidepsin to manipulate Tax expression and provide a foundation for further research into eliminating latently infected cells. These findings also contribute to a better understanding of conditions limiting transcription and translation of viral gene products.

## Introduction

Human T-cell leukemia virus type 1 (HTLV-1) is an oncogenic δ-retrovirus that infects five to ten million people worldwide. However, this is probably an underestimation since epidemiological data are lacking for several highly-populated areas (1–3). Infection with HTLV-1 is associated with several diseases, including malignant and inflammatory conditions. Around five percent of all infected individuals develop adult T-cell leukemia/lymphoma (ATL). However, in carriers infected by mother-to-child transmission, it is estimated that the risk rises to 25% (3). HTLV-1-associated myelopathy/tropical spastic paraparesis (HAM/TSP) occurs in one to four percent of infected people, depending on ethnicity (4). HTLV-1 infection also leads to a range of other inflammatory diseases, including uveitis, arthropathy, and dermatitis (3, 5). It has been recognized recently that “asymptomatic carriers” of HTLV-1 also have an impaired quality of life and a reduced life expectancy (6). The prognosis of both ATL and HAM/TSP is dismal, and there is a clear need for new therapeutic options (2). HTLV-1 predominantly infects CD4^+^ T-cells and, after integration into the host genome, enters a state of clinical latency (7). The resulting polyclonal reservoir of HTLV-1-infected cells proves to be the main obstacle in eliminating infected cells, as in human immunodeficiency virus type 1 (HIV-1) infection. The kick-and-kill or shock-and-kill approach is a strategy that has been tested to mitigate the infection and to reduce the size of the HIV-1 reservoir. The aim of this approach is to reactivate sense-strand expression of the provirus to expose it to the host’s immune response, thereby enabling eradication of infected cells (8–11).

A central mechanism in silencing transcription in the context of latency is chromatin remodeling mediated by structural modification of histones. One of the histone alterations that contributes to repressed gene expression is hypoacetylation of chromatin, which is catalyzed by histone deacetylases (HDACs). HDACs are grouped into four classes, differing in their localization and functional properties. Classical classes include classes I, II, and IV, which rely on zinc as a cofactor. Class I HDACs (HDAC 1, 2, 3, 8) constitutively localize in the nucleus and are ubiquitously expressed in the human body. Class II HDACs are further divided into class IIa (HDAC 4, 5, 7, 9) and IIb (HDAC 6, 10). They are expressed tissue-specifically in the body and shuttle between the nucleus and the cytoplasm. The sole member of class IV is HDAC 11, which combines class I and class II properties (12–14). While class I, II, and IV can be inhibited by histone deacetylase inhibitors (HDACi), class III HDACs are functionally distinct and are unaffected by HDACi, relying on NAD^+^ as a cofactor (15, 16).

HDACi are a promising group of latency-reversing agents (LRAs) to induce chromatin hyperacetylation, generally associated with transcriptional activation and enhanced gene expression. Consequently, these compounds are most often studied to reactivate the latent viral reservoir in the kick-and-kill approach (12). Based on their cytotoxicity, their potency, and their selectivity towards inhibited HDACs, HDACi are further divided into (1) hydroxamates (e.g. Panobinostat), (2) benzamides (e.g. Entinostat), (3) short-chain fatty acids (e.g. Valproate), (4) cyclic peptides (e.g. Romidepsin), and (5) selective inhibitors (e.g. MC1568) (17). Hydroxamates and cyclic peptides are very potent HDACi. Short-chain fatty acids display poor bioavailability and, together with benzamides, are less potent (18, 19).

Earlier work has tried to translate the kick-and-kill approach to HTLV-1 infection, employing HDACi to activate viral gene expression (20, 21). HTLV-1 transcription is tightly regulated, and is most potently activated by the viral transactivator protein Tax. Tax is encoded by the sense-strand of HTLV-1 and is essential for the initiation of malignant transformation of infected T-cells (22). Tax is also the immunodominant target of cytotoxic T-cells (CTLs) since chronically activated CTL responses directed against Tax are present in HTLV-1 infected people (23, 24). It has been possible to transiently increase Tax expression in kick-and-kill trials, resulting in a temporary decrease in the proviral load (PVL). However, these studies failed to achieve sustained repression of the PVL (20, 21, 25). There is evidence that distinct HDAC complexes form at the HTLV-1 long terminal repeats (LTRs). It has been shown that HDAC 1 and 2 predominantly bind at the 5’LTR, whereas HDAC 3 favors the 3’LTR (26). Since previous *in vivo* and *ex vivo* studies were limited to the class I and class IIa HDACi Valproate, a member of the short-chain fatty acid HDACi, we reasoned that more potent or more selective HDACi might be better suited to reverse the latency of HTLV-1.

In this study, we systematically compared a panel of different HDACi for their potential to activate HTLV-1, represented by Tax expression, in different model systems *in vitro*. We identified the pan-HDACi Panobinostat and the class I HDACi Romidepsin as the most potent HDACi for HTLV-1 reactivation. We conclude that these agents hold great potential as latency-reversing agents in strategies aimed at reducing the size of the HTLV-1 reservoir.

## Materials and methods

### Cell lines and patient samples

The CD4^+^ Jurkat T-cells (27) were cultured in RPMI 1640 (Thermo Fisher Scientific, Waltham, USA) supplemented with 45% Panserin 401 (PAN-Biotech GmbH, Aidenbach, Germany), 10% fetal calf serum (FCS) (Capricorn Scientific GmbH, Ebsdorfergrund, Germany), L-glutamine (0.35 g/l) (GlutaMAX™ from Thermo Fisher Scientific) and penicillin/streptomycin (0.12 g/l each) (Thermo Fisher Scientific). The HTLV-1 positive *in vitro* transformed CD4^+^ MT-2 cells (28) were cultured in RPMI 1640 containing 10% FCS, L-glutamine, and penicillin/streptomycin.

Peripheral blood mononuclear cells (PBMCs) were obtained from blood samples from HTLV-1 infected patients attending the National Centre for Human Retrovirology at St Mary’s Hospital (Imperial College Healthcare NHS Trust, London, UK). Patients are invited to participate in the Communicable Diseases Research Tissue Bank (CDRTB) of Imperial College London and following written informed consent donate blood samples when attending for routine clinical care. The CDRTB is approved by the National Research Ethics Service (reference 20/SC/0226). HTLV-1 proviral load is determined at each patient visit by real-time PCR as previously described (29). PBMCs were cryopreserved in FCS supplemented with 10% dimethylsulfoxide (DMSO) at -150°C until use. After thawing, PBMCs were washed in Dulbecco’s Balanced Salt Solution (without calcium or magnesium) (DPBSo) (Thermo Fisher Scientific). CD4^+^ T-cells were isolated *via* immunomagnetic negative selection with the EasySep™ Human CD4+ T Cell Isolation Kit (STEMCELL Technologies, Vancouver, Canada). During isolation, cells were incubated with 20 µl/ml of Isolation Antibody Cocktail for 10 min. Afterward, cells were incubated with 40 µl/ml of the Dextran RapidSpheres for 5 min. After another washing step in PBSo, PBMCs were cultivated in RPMI 1640 supplemented with 20% FCS, L-glutamine, penicillin/streptomycin, 5.5 mM glucose, and 10 µM Raltegravir (solved in DMSO) (Selleckchem, Houston, USA).

### Plasmids and transfection

Jurkat T-cells were transiently transfected *via* electroporation. To this end, a Gene Pulser Xcell^®^ Electroporation System (Bio-Rad, Hercules, USA) was used. Briefly, 5*10E6 cells and 50 µg of total plasmid DNA were suspended in 0.8 ml cell culture media without penicillin/streptomycin. Electroporation was performed in electroporation cuvettes with an electrode distance of 4 mm (Peqlab, Erlangen, Germany) at 290 V and 1500 µF. Cells were harvested at 48 h after transfection for further analysis. For Tax expression in Jurkat T-cells, 15 µg of the plasmid LTR-Tax (pLcXL) (30) carrying the Tax expression cassette under the control of the HTLV-1 LTR was transiently transfected. The plasmids pEF1α and pcDNA3.1 (both Thermo Fisher Scientific) served as empty vector control. Furthermore, 10 µg of pGL3-U3R-Luc, a luciferase reporter vector containing a *firefly luciferase* gene under the control of HTLV-1 core promoter *U3R* (31), was transfected.

### Chemical treatment

In the case of prior transfection, cells were pooled and reseparated 24 h after transfection. The HDACi used for treatment were Belinostat (PXD101/Beleodaq; 100 nM to 10 µM; Sigma-Aldrich, St. Louis, USA), Entinostat (MS-275; 10 nM to 10 µM; Selleckchem), MC1568 (1 µM to 100 µM; Tocris, Bristol, UK), Panobinostat (LBH589/Farydak; 10 nM to 10 µM; Selleckchem), Romidepsin (FK228/Istodax; 10 nM to 1 µM; Selleckchem), Valproate (50 µM to 3 mM; Sigma-Aldrich) and Vorinostat (SAHA/Zolinza; 100 nM to 5 µM; Selleckchem). The topoisomerase II inhibitor Etoposide (15 µM; Sigma-Aldrich) served as toxicity control. A combination of Phorbol 12-myristate 13-acetate (PMA; 20 nM; Cell Signaling Technology, Cambridge, UK) and Ionomycin (1 µM; Merck, Darmstadt, Germany) was employed as a positive control for the induction of viral gene expression. All of the compounds mentioned above were dissolved in DMSO, except for Valproate, which was dissolved in water. The volume added to the cell culture during chemical treatment was equivalent to one percent of the culture volume.

### Lactate dehydrogenase (LDH) release assay

Cytotoxic effects of the compounds were assessed with the LDH-Glo™ Cytotoxicity Assay (Promega, Fitchburg, USA) following the manufacturer’s instructions. Briefly, the supernatant was collected from 500,000 cells per sample 24 h after chemical treatment, diluted 1:20 in LDH storage buffer, and stored at -20°C until further analysis. For the LDH release assay, a technical or biological duplicate of the supernatant was diluted to a final concentration of 1:100 in LDH storage buffer (10 µl from 1:20 dilution in 40 µl storage buffer) and one part (50 µl) of the LDH detection reagent was added. After one hour of incubation, luminescence was recorded on a Victor X4 (PerkinElmer, Waltham, USA). A maximum LDH release control was performed by adding 2 µl of 10% Triton X-100 (Triton) per 100 µl cell culture volume 15 min before collecting the supernatant. The luminescence of the samples was normalized to untreated or empty vector and Triton to calculate cytotoxicity.

### Quantitative real-time RT-PCR

For isolation of mRNA from Jurkat T-cells, the NucleoSpin^®^ RNA Kit (Macherey-Nagel, Düren, Germany) was used. To isolate mRNA from MT-2 cells, the RNeasy Mini Kit (Qiagen, Venlo, Netherlands) in combination with QIAshredder columns (Qiagen) was used. To isolate RNA from primary cells, the RNeasy Micro Kit (Qiagen) combined with QIAshredder columns was used. cDNA preparation from up to 5 µg of isolated RNA was performed with the SuperScript™ II Reverse Transcriptase (Thermo Fisher Scientific) combined with Random Hexamer Primers (Thermo Fisher Scientific) per manufacturer’s instructions. A technical triplicate of 200 ng of the obtained cDNA was subjected to quantitative real-time RT-PCR (qRT-PCR), employing the TaqMan^®^ Universal PCR Master Mix and a 7500 Real-Time PCR System (both Thermo Fisher Scientific). The primer/probe pairs and TaqMan Gene Expression Assays (Thermo Fisher Scientific) used to detect transcripts are listed in **Table 1**. **Table 1** also lists the plasmids used to generate standard curves allowing for the quantification of transcripts. Data were evaluated with the 7500 Software v2.3 (Thermo Fisher Scientific). Every experiment was independently performed at least three times, and relative copy numbers (rcn) were calculated by normalization of respective transcript levels on those of *β-actin*.

**Table 1:** Sequences of oligonucleotides for qRT-PCR analysis.

Gene	Type	Sequence (5’-3’) or Assay ID	Reference
4-1BB	4-1BB fwd rt	TGGCTGTAGCTGCCGATTTC	(32)
	4-1BB rev rt	AAAGTCCCAACAGCCCTATTGA	
	4-1BB probe	FAM-CTTCCATTTCACAGTTCACATCCTCCTTCTTC T-TAMRA	
	Std	pJET_4-1BB	
β-actin	β-actin fwd rt	CCTCGCCTTTGCCGA	(32, 33)
	β-actin rev rt	TGGTGCCTGGGGCG	
	β-actin probe	FAM-CCGCCGCCCGTCCACACCCGCC-TAMRA	
	Std	pJET_ACTB	
FOXP3	AoD	Hs00203958_m1	(34), Addgene #153147
	Std	Flag-FOXP3	
gag	gag fwd rt	AGCCCCCAGTTCATGCAGACC	(35, 36)
	gag rev rt	GAGGGAGGAGCAAAGGTACTG	
	gag probe	FAM-CTGCCAAAGACCTCCAAGACCTCC-TAMRA	
	Std	pCMVHT1M	
HIAP-1	AoD	Hs00154109_m1	(37)
	Std	pcDNA3.1-HIAP1	
sHBZ	sHBZ fwd rt	CTTCTAAGGATAGCAAACCGTCAAG	(35, 38, 39)
	sHBZ rev rt	ATGGCGGCCTCAGGGCT	
	sHBZ probe	FAM-CCTGTGCCATGCCCGGAGGA-TAMRA	
	Std	pcDNA3.1-HBZ-wt-MycHis	
tax	tax fwd rt	TGGCCCATTTCCCAGGGTTTG	New
	tax rev rt	GAGTCGAGGGATAAGGAAC	(40)
	tax probe	FAM-TACAAGGCGACTGGTGCC-TAMRA	(41)
	Std	pcTax	(42)

fwd, forward primer; rt, real time; rev, reverse primer; FAM, 6-carboxyfluorescein; TAMRA, tetramethylrhodamine; std, plasmid used for the respective standard curve; AoD, TaqMan Gene Expression Assays (Thermo Fisher Scientific).

### Western blot

For Western Blot analysis, three to five million cells were harvested, washed in PBSo, and lysed in lysis buffer [150 mM NaCl, 10 mM Tris/HCl (pH 7.0), 10 mM EDTA, 1% Triton X-100, 2 mM DTT and protease inhibitors leupeptin, aprotinin (20 μg/ml each) and 1 mM phenylmethylsulfonyl fluoride (PMSF)]. After cell lysis, samples were subjected to two freeze-and-thaw cycles to facilitate lysis, consisting of at least 5 min freezing in liquid nitrogen and subsequent thawing at 30°C, 1400 rpm for 3 min. After that, sonification was carried out in a Branson 450 Sonifier (Emerson, Ferguson, USA) with the output control 8 and 80% duty cycle at 4°C for 30 seconds five times. After sonification, the cell detritus was pelleted at 20,817 relative centrifugal force (rcf) for 15 to 70 min to obtain a purified protein lysate. According to the manufacturer’s protocol, protein concentrations of the lysates were determined with ROTI^®^Quant (Carl Roth, Karlsruhe, Germany), and 40 µg to 50 µg of protein were denatured in sodium dodecyl sulfate (SDS) loading dye [10 mM Tris/HCl (pH 6.8), 10% glycerin, 2 % SDS, 0.1 % bromophenol blue, 5 % β-mercaptoethanol] at 95°C for 10 min. Samples were run at 130V on SDS-PAGE gels in XCell Sure Lock Mini Western Blot chambers (Thermo Fisher Scientific), and proteins were subsequently blotted onto Amersham Protran nitrocellulose membranes (GE Healthcare, Chicago, USA) at 250 mA for 75 min. PageRuler Prestained Protein Ladder (Thermo Fisher Scientific) was used as a molecular weight marker.

After blocking of membranes (1x TBS, 0.1% Tween-20, 5% FCS) for one hour at room temperature, staining with the following primary antibodies was performed for one hour at room temperature or overnight at 4°C: rabbit polyclonal anti-acetyl-Histone H3 Antibody (Ac-H3) (06-599, Millipore, Merck, Darmstadt, Germany), mouse anti-Tax (derived from the hybridoma cell line 168B17-46-34) (43), and mouse monoclonal anti-α-Tubulin clone DM1A (Tubulin) (T9026, Sigma-Aldrich). Subsequently, incubation with the secondary antibodies, conjugated with horseradish peroxidase (GE Healthcare, Chicago, USA), was performed at room temperature for 30 min. Between incubation with different antibodies, the membranes were washed in washing buffer (1x TBS, 0.1% Tween-20) for three times ten minutes between incubation periods. Enhanced chemiluminescence of the blots was recorded on an Intas Advanced Imager camera (Intas Science Imaging Instruments GmbH, Göttingen, Germany). Densitometric analysis of Tax, Ac-H3, and Tubulin protein bands was performed with the Advanced Image Data Analyzer (AIDA Version 4.23.035, Elysia-raytest GmbH, Straubenhardt, Germany). Mathematical values smaller than zero were corrected to zero to evaluate the densitometric analysis.

### Flow cytometry

Different staining protocols were performed for MT-2 cells and primary cells. For detection of apoptosis and cell death, MT-2 cells were stained with the LIVE/DEAD Fixable Far Red Dead Cell Stain Kit (Thermo Fisher Scientific) for 30 min. Thereafter, Annexin V staining was carried out with the Annexin V pacific blue conjugate (Thermo Fisher Scientific) in 100 µl of 1x Annexin Binding Buffer (Thermo Fisher Scientific) for 15 min. For acquisition, cells were resuspended in 1x Annexin Binding Buffer. In MT-2 cells, intracellular Tax was stained with the Inside Stain Kit (Miltenyi Biotec, Bergisch Gladbach, Germany) in combination with the mouse anti-Tax (derived from the hybridoma cell line 168B17-46-34) (43) and the Alexa Fluor^®^ 647 α-mouse antibody (Thermo Fisher Scientific). eBioscience™ Mouse IgG1 κ Isotype Control Clone P3 (Thermo Fisher Scientific) was used as isotype control.

To evaluate the viability of primary cells, the LIVE/DEAD Fixable Near-IR Dead Cell Stain Kit (Thermo Fisher Scientific) was employed for 15 min. Subsequently, extracellular CD4 was stained with mouse FITC anti-human CD4 clone RPA-T4 (BioLegend, San Diego, USA) in PBSo supplemented with 7% FCS for 30 min. In the next step, primary cells were stained with the eBioscience™ Foxp3/Transcription Factor Staining Buffer Set (Thermo Fisher Scientific) combined with the anti-Tax AF647 clone Lt-4 (44). Alexa Fluor^®^ 647 IgG3, κ clone MG3-35 (BioLegend) was used as isotype control.

All incubation steps were carried out at room temperature and protected from light. After each staining step, cells were washed at least once in PBSo, a FACS buffer (PBSo supplemented with 2-7 % FCS and 2 mM EDTA), or permeabilization buffer of the intracellular staining kits. Regularly, cells were resuspended in 200 µl to 400 µl of PBSo for flow cytometry. Samples were measured with a BD LSR II or BD LSRFortessa flow cytometer (BD, Franklin Lakes, USA) and recorded with the BD FACSDiva™ software (BD). Further data analysis was carried out with FCS Express Version 3 (*De Novo* Software, Los Angeles, USA) for the chronically infected cells and FlowJo 10.8.1 (BD) for the primary cells. Doublets were excluded during the analysis.

### Gag p19 ELISA

Cell culture supernatant was collected and stored at -80°C. For further analysis, the supernatant was thawed, passed through sterile filters (pore size 0.45 µm), and processed as a technical duplicate according to the HTLV p19 Antigen ELISA manual (ZeptoMetrix, Buffalo, USA). Fluorescence was recorded on a Victor X4.

### Statistics

For statistical analysis, Student’s t-test (unpaired, two-tailed) and Pearson’s correlation were performed as indicated using Microsoft Office Excel. P values < 0.05 were considered as significant (*).

## Results

### Most HDACi only mildly affect cell viability

**Figure 1:**
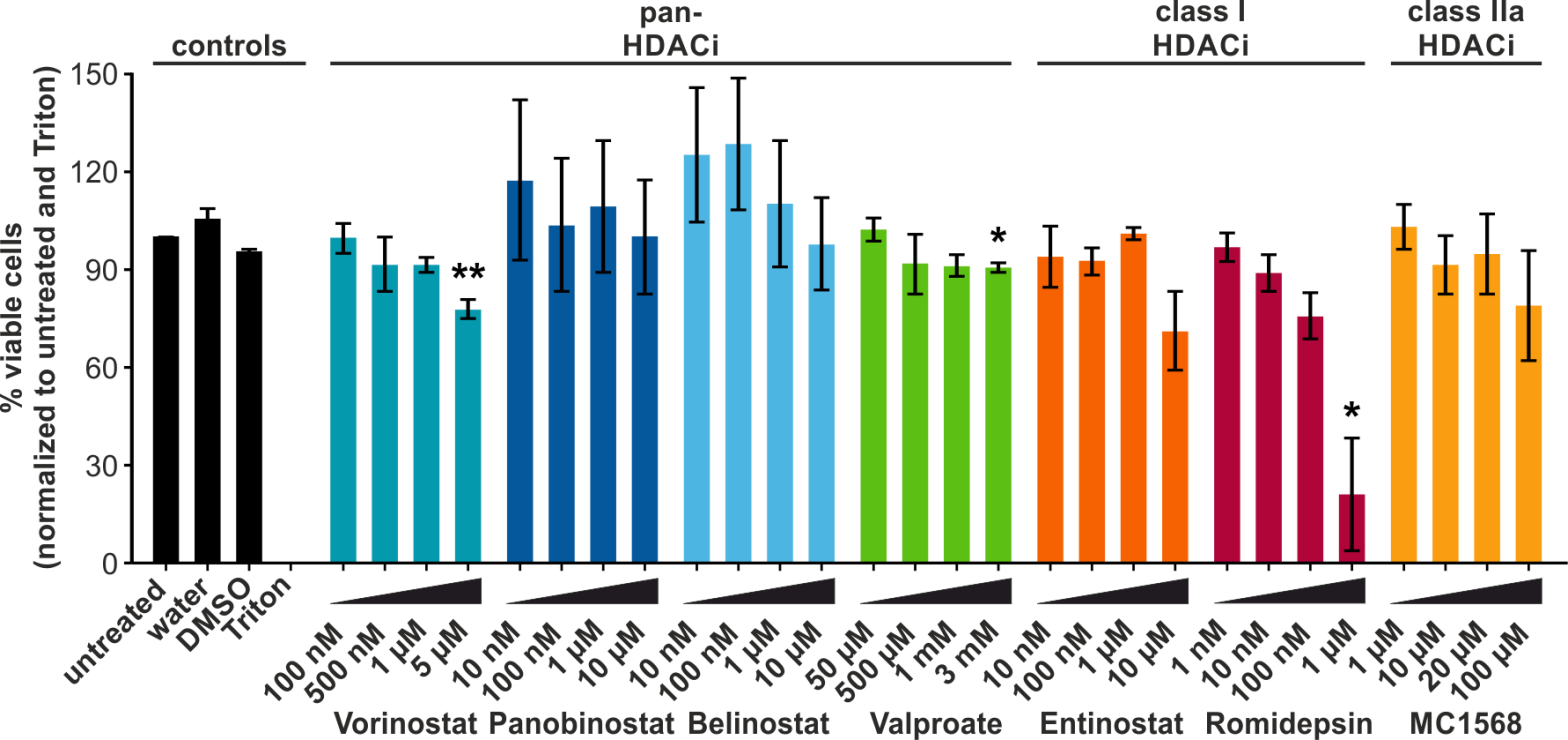
HDACi only mildly impair the viability of Jurkat T-cells. LDH release assays were performed in the Jurkat-U3R-Luc T-cells after 24 h of chemical treatment with pan-HDACi (Vorinostat, Panobinostat, Belinostat, Valproate), class I HDACi (Entinostat, Romidepsin), or class IIa HDACi (MC1568). Cells treated with Triton (dead cells) and untreated were set as 0% and 100 % viable cells, respectively. DMSO served as solvent control for all HDACi, except Valproate. Mean values of three independent experiments ± standard error (SE) are depicted and were compared using Student’s t-test (*, p < 0.05, or **, p < 0.01 relative to respective solvent control).

LRAs are commonly used in attempts to reactivate transcription of retroviruses, such as HTLV-1 (45). *Ex vivo* and *in vivo* approaches in HTLV-1 infection have focused primarily on the short-chain fatty acid Valproate. However, despite a transient decrease in the PVL of HAM/TSP patients, Valproate treatment failed to achieve a permanent reduction (20, 21, 25). To identify LRAs which are more potent than Valproate at modulating or reversing the latency of HTLV-1, we screened a number of additional HDACi: (1) Vorinostat, Panobinostat, Belinostat, and Valproate (all pan-HDACi, except Valproate being specific for class I and IIa, but often simplified as pan-HDACi), (2) Entinostat, and Romidepsin (both class I HDACi), and (3) MC1568 (class IIa HDACi). The selected HDACi were representative of the structural classes commonly used to categorize HDACi. HDACi of the hydroxamate group, Vorinostat, Panobinostat, and Belinostat (blue bars, **Figure 1**), the short-chain fatty acid Valproate (green bars), the benzamide and class I HDACi Entinostat (orange), the cyclic peptide HDACi Romidepsin (pink), and the class IIa specific HDACi MC1568 (yellow) were analyzed. First, we examined the cytotoxicity of the selected HDACi. To this end, Jurkat T-cells were treated for 24 h with different concentrations of the compounds, and cytotoxicity was assessed *via* an LDH release assay. LDH functions as a marker of cell damage. HDACi treatment only mildly impaired cell viability, which remained above 75% in most cases. Exceptions to this were the exposure to 10 µM Entinostat or 1 µM Romidepsin, resulting in 71% and 21% viable cells, respectively. The decrease in viability was only statistically significant in the case of the exposure to 5 µM Vorinostat (78% viable cells), 3 mM Valproate (91% viable cells), and 1 µM Romidepsin (21 % viable cells). Based on these results, suitable treatment concentrations for further experiments investigating the functionality of these HDACi were selected that align with previous findings (20, 46–48).

### HDACi increase Tax protein to a lesser extent than *tax* transcripts in transiently transfected cells

**Figure 2:**
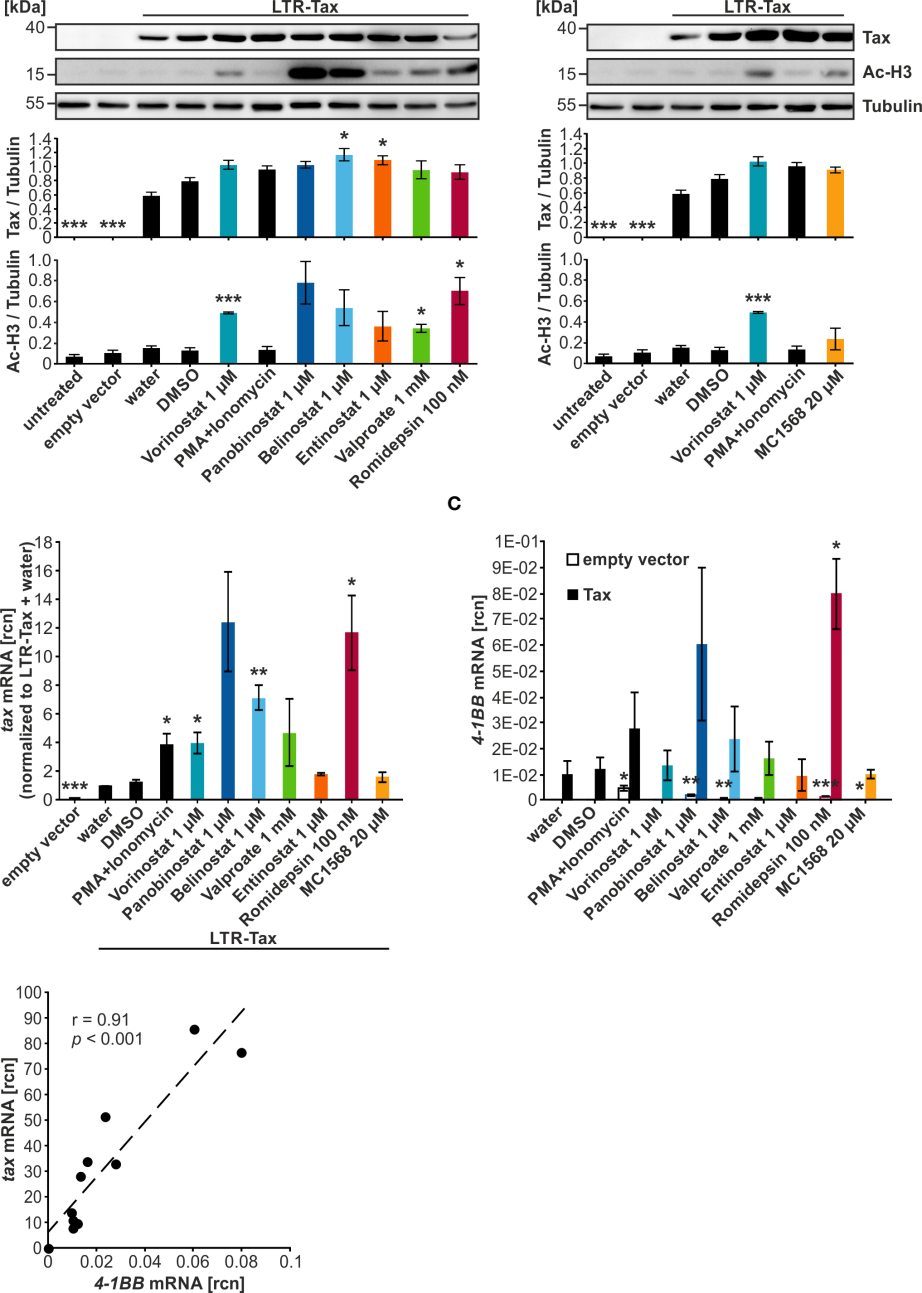
HDACi significantly enhance *tax* transcription, but only mildly Tax protein expression. **(A–D)** Jurkat T-cells were transiently transfected with 15 µg of the plasmid LTR-Tax and filled with the empty vector DNA pEF1α to 50 µg. At 24 h after transfection, chemical treatment was carried out for 24 h. PMA+Ionomycin served as a positive control for activation of viral transcription. **(A)** Western Blot shows Tax protein, acetylated histone H3 (Ac-H3), and α-Tubulin (Tubulin) as the loading control. Densitometric analysis of Tax protein expression, and Ac-H3, relative to Tubulin, of three independent experiments, was performed. Mean values of three independent experiments ± SE are depicted and were compared using Student’s t-test (*p < 0.05 relative to respective solvent control). **(B)** Fold change of relative copy numbers (rcn) of *tax*, normalized to the housekeeping gene *β-Actin* and LTR-Tax and water, of three independent experiments ± SE is displayed and was compared using Student’s t-test (*p < 0.05; **p < 0.01; ***p < 0.001, relative to the respective solvent control; empty vector was compared to LTR-Tax and water). **(C)** Mean rcn of *4-1BB*, normalized to the housekeeping gene *β-Actin*, of three independent experiments ± SE was compared to the respective solvent control-treated cells using Student’s t-test (*p < 0.05; **p < 0.01; ***p < 0.001) in empty vector (clear outlined bars) or LTR-Tax-transfected (black and colored bars) Jurkat T-cells. **(D)** Mean rcn of *tax* and *4-1BB*, normalized to the housekeeping gene *β-Actin*, were correlated and subjected to Pearson correlation analysis (Pearson correlation coefficient r = 0.91; *p* = 6.9E-05).

To screen the selected HDACi for their ability to enhance protein and transcript levels of the HTLV-1 viral transactivator Tax, Jurkat T-cells were transiently transfected with the plasmid LTR-Tax carrying the Tax-expression cassette under the control of the natural LTR promoter sequence, and cells were treated for 24 h with the HDACi in the indicated concentrations (**Figure 2A**). To avoid missing of potential effects, the highest concentrations of compounds were selected that resulted in at least 75% viable cells (**Figure 1**). The combination treatment of PMA and Ionomycin served as a positive control for activation of transcription. Western blot analysis demonstrated that all tested HDACi and the positive control PMA+Ionomycin achieved raised Tax protein levels above their respective solvent controls (**Figure 2A**). However, this increase remained moderate and was only statistically significant in the case of Belinostat and Entinostat as detected by densitometry (**Figure 2A**). As expected, treatment with the HDACi also resulted in intensified levels of acetylation of histone H3 (Ac-H3), resulting in less condensed chromatin, which is more likely to be transcribed (49). The rise in Ac-H3 caused by Panobinostat, Belinostat, and Romidepsin was very pronounced. Yet, a statistically significant increase was only observed after treatment with Vorinostat, Valproate, and Romidepsin (**Figure 2A**).

In order to explore the effect of the HDACi on *tax* transcript levels, reverse transcriptase quantitative PCR (qRT-PCR) was used to determine relative copy numbers (rcn) of *tax* (**Figure 2B**) and the Tax-target gene *4-1BB* (32), a costimulatory receptor of the tumor necrosis factor receptor superfamily (**Figure 2C**). qRT-PCR is more sensitive than Western Blot, and the effects of the LRAs appeared to be more pronounced at the mRNA level. All tested agents and the positive control PMA+Ionomycin raised *tax* transcription above their respective solvent control. This enhancement was significant when the cells were treated with PMA+Ionomycin, Vorinostat, Belinostat, or Romidepsin. However, a particularly profound increase was only seen in the case of Panobinostat, Belinostat, and Romidepsin. Analysis of the Tax-target gene *4-1BB* revealed that Romidepsin raised *4-1BB* transcription significantly above the solvent control level. Panobinostat overall also displayed enhanced *4-1BB* expression, but this change was not significant. Pearson correlation analysis confirmed a highly significant direct correlation between *tax* and *4-1BB* mRNA (**Figure 2D**; r = 0.91; *p* = 6.9E-05). A comparison of *4-1BB* mRNA levels after HDACi treatment between LTR-Tax and empty vector-transfected cells demonstrated that the substantial increase in transcription was specific to the LTR-Tax-transfected cells and was not solely a result of the compound administration (**Figure 2C**; **Supplementary Figure S1**). We conclude that HDACi are promising agents to activate HTLV-1 transcription. These findings are in agreement with previous studies, which showed that Vorinostat, Panobinostat, Valproate, and Romidepsin can be used to reverse HTLV-1 latency *in vitro* (20, 50, 51). However, our data suggest that Panobinostat, Belinostat, and Romidepsin might be more successful at causing an enduring and extensive manipulation of Tax expression than Valproate.

### Dose-dependent effects of Panobinostat, Belinostat, and Romidepsin on Tax protein and *tax* transcripts in transiently transfected cells

**Figure 3:**
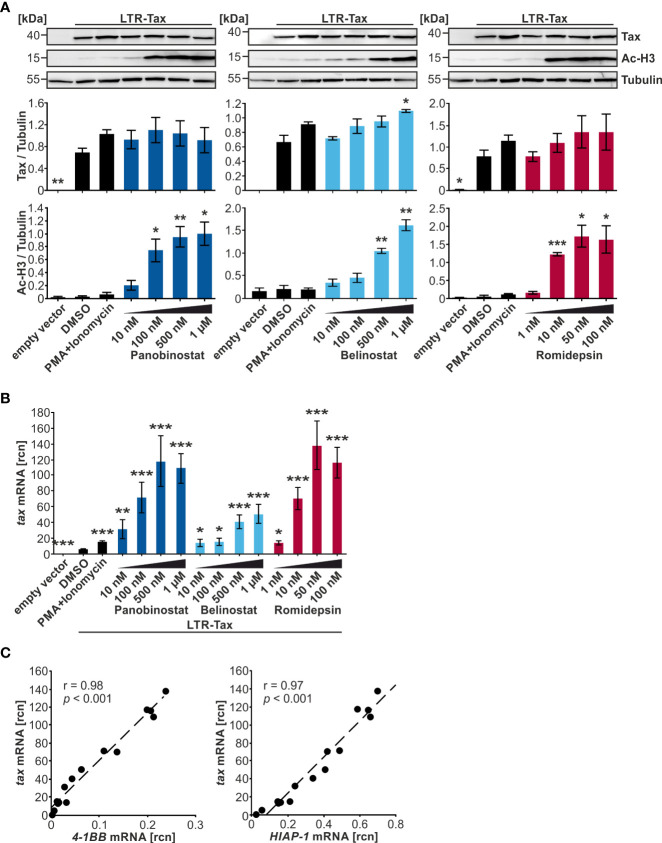
Despite a strong dose-dependent increase in histone acetylation, Tax protein is only slightly enhanced by HDACi in Jurkat T-cells. **(A–C)** Jurkat T-cells were transiently transfected with 15 µg LTR-Tax and filled with the empty vector DNA pEF1α to 50 µg. Increasing doses of HDACi Panobinostat or Belinostat (10 nM, 100 nM, 500 nM, 1 µM), or Romidepsin (1nM, 10 nM, 50 nM, 100 nM) were added at 24 h post transfection for 24 h. PMA+Ionomycin served as positive control for activation of viral transcription and DMSO as solvent control. **(A)** Western Blot shows Tax protein, acetylated histone H3 (Ac-H3), and α-Tubulin (Tubulin) as the loading control. Densitometric analysis of Tax protein expression and Ac-H3, relative to Tubulin, of three independent experiments was performed. Mean values ± SE are depicted and HDACi treatment was compared to DMSO treatment using Student’s t-test (*p < 0.05; **p < 0.01; ***p < 0.001). **(B)** Mean rcn of *tax* normalized to those of the housekeeping gene *β-Actin* of three independent experiments, and of nine independent experiments for the controls, ± SE are depicted, and values were compared to DMSO using Student’s t-test (*p < 0.05; **p < 0.01; ***p < 0.001). **(C)** Mean rcn of *tax* and the Tax-target genes *4-1BB* and *HIAP-1*, normalized to the housekeeping gene *β-Actin*, were correlated and subjected to Pearson correlation analysis (Pearson correlation coefficient r = 0.98 and r = 0.97 respectively, *p* = 9.05E-12 and *p* = 2.90E-10 respectively).

Due to the promising performance of Panobinostat, Belinostat, and Romidepsin in raising *tax* transcription in transiently transfected Jurkat T-cells, these three HDACi were selected for further evaluation and dose-finding experiments in the same model system. Analogous to the preceding experiments, Jurkat T-cells were transfected with LTR-Tax and incubated with four concentrations of the compounds for 24 h. An LDH release assay revealed that Jurkat T-cell viability was significantly reduced by the upper concentrations of Panobinostat (100 nM, 500 nM, 1 µM), Belinostat (500 nM, 1 µM), and Romidepsin (50 nM, 100 nM) (**Supplementary Figure S2A**) in the presence of Tax, compared to cells were Tax was absent (**Figure 1**). However, independent of the concentration tested, at least 80% of cells were still viable upon treatment (**Supplementary Figure S2A**), thus allowing further analysis. All HDACi tested induced acetylation of histone H3 dose-dependently, as detected by anti-Ac-H3 antibodies (**Figure 3A**). Minimal dosages required for a significant increase in Ac-H3 were 100 nM Panobinostat, 500 nm Belinostat and 10 nM Romidepsin, respectively. Despite a strong rise in Ac-H3 between the two lowest concentrations of Panobinostat and Romidepsin, the further increase in acetylation at the highest employed concentration was less prominent: a maximum level of achievable acetylation appeared to have been reached. Concomitantly, Panobinostat, Belinostat, and Romidepsin led to a dose-dependent enhancement of *tax* mRNA, which was significant in all of the tested concentrations (**Figure 3B**). However, 100 nM Panobinostat and 10 nM Romidepsin were more potent than 1 µM Belinostat (**Figure 3B**). The increase in *tax* mRNA (**Figure 3B**) correlated well with that of Ac-H3 (**Figure 3A**) (Pearson correlation r = 0.88; *p* = 1.40E-07; **Supplementary Figure S2C**), linking the mode of action of the HDACi closely to the activation of transcription. However, despite this observed induction of transcription, the Tax protein level did not change much compared to the solvent control (**Figure 3A**). Only one concentration of Belinostat resulted in a significant increase of Tax protein. Additionally, there is no dose-dependency visible in the increase of Tax protein upon Panobinostat treatment. The inadequate representation of increased *tax* transcription in Tax protein expression was mirrored by a weaker correlation between *tax* mRNA and Tax protein levels (Pearson correlation r = 0.63; *p* = 2.1E-03; **Supplementary Figure S2C**), as well as between Ac-H3 and Tax protein (Pearson correlation r = 0.62; *p* = 2.7E-03; **Supplementary Figure S2C**). The failure of Tax protein expression to reflect raised *tax* transcription points toward additional barriers in Tax expression posing as a post-transcriptional block, which has also been described for the related retrovirus HIV-1 (52).

Expression of the NF-κB-dependent Tax-target genes *4-1BB* and *HIAP-1* was also measured; the latter acts as an inhibitor of apoptosis by binding caspases 3, 7, and 9 (37). Both *4-1BB* and *HIAP-1* mRNA displayed a highly significant positive Pearson correlation with *tax* mRNA (**Figure 3C**; r = 0.98 and r = 0.97 respectively; *p* = 9.05E-12, and *p* = 2.90E-10 respectively; **Supplementary Figure S2B**, upper and middle panel), suggesting that either *tax* mRNA is able to induce NF-κB-dependent target genes, or that Tax protein was induced by HDACi treatment, but at undetectable levels or at an earlier timepoint. To investigate whether the boost in transcription following HDACi treatment is specific for *tax* and Tax-target genes, mRNA levels of the non-Tax-target gene *FOXP3* were measured, whose expression is upregulated by HBZ, the sole anti-sense-strand product of HTLV-1 (53). Transfection of LTR-Tax and treatment with the solvent control DMSO led to slightly lower levels of *FOXP3* than in the empty vector control, underscoring that Tax does not enhance *FOXP3* transcription (**Supplementary Figure S2B**, lower panel). HDACi treatment raised *FOXP3* mRNA levels. Nevertheless, the absolute rcn of *FOXP3* remained two orders of magnitude beneath the rcn of the Tax-target genes at a level that we would not consider physiologically relevant (**Supplememtary Figure S2B**, lower panel). These findings demonstrate that while Panobinostat, Belinostat, and Romidepsin significantly induce transcription of *tax* and Tax target genes, Tax protein levels remain largely unaffected. Finally, out of the group of hydroxamate HDACi, Panobinostat is more potent than Belinostat in this setting at inducing *tax* transcription.

### Selected HDACi dose-dependently induce apoptosis in chronically infected cells

**Figure 4:**
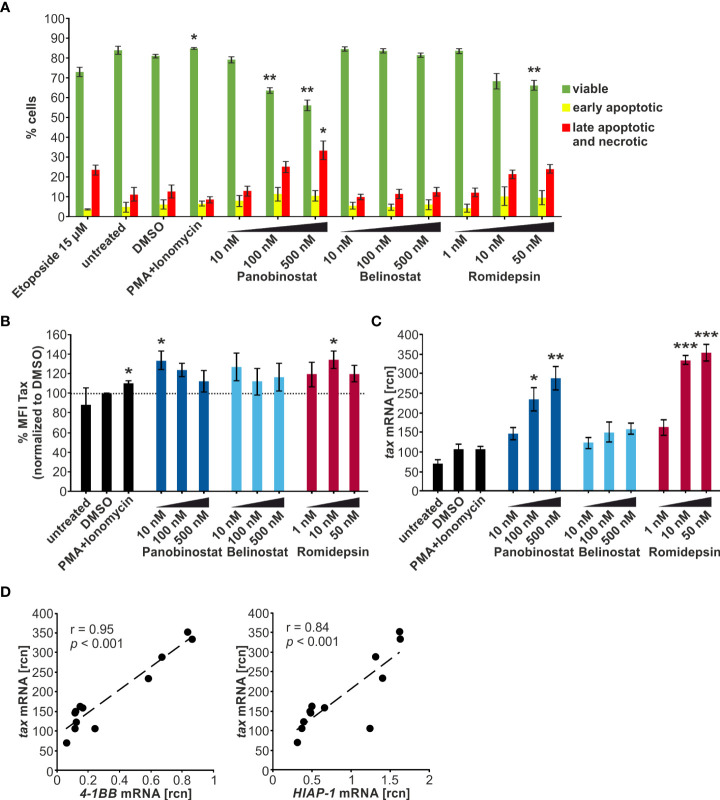
Panobinostat, Belinostat, and Romidepsin dose-dependently increase *tax* transcripts, but only mildly Tax protein in chronically HTLV-1-infected cells. **(A–D)** MT-2 cells were treated with increasing concentrations of the HDACi Panobinostat, Belinostat, or Romidepsin as indicated for 24 h. PMA+Ionomycin served as a positive control for activation of latent viral transcription, DMSO served as solvent control. Cells were subjected to **(A, B)** flow cytometry and **(C, D)** qRT-PCR. **(A)** Flow cytometry upon staining of cells with the LIVE/DEAD Fixable Far Red Dead Cell Stain Kit and the Annexin V pacific blue conjugate. The frequencies of viable cells (double-negative cells; green), early apoptotic cells (LIVE/DEAD^-^/Annexin V^+^; yellow), or late apoptotic and necrotic cells (LIVE/DEAD^+^; red) are indicated (gating strategy: **Supplementary Figure 3A**). Etoposide (15 µM) served as a positive control for induction of apoptosis. Mean percentages of each cell population of three independent experiments ± SE are depicted and were compared to DMSO treatment using Student’s t-test (*, *p* < 0.05; **, *p* < 0.01). **(B)** Flow cytometry of Tax protein in living cells. Fold change of arithmetic mean fluorescence intensities normalized to DMSO of three independent experiments ± SE are depicted, and values were compared to DMSO treatment using Student’s t-test (*, *p* < 0.05). **(C)** Mean rcn of *tax* normalized to the housekeeping gene *β-Actin*, of four independent experiments ± SE were compared to DMSO-treated cells using Student’s t-test (*, *p* < 0.05; **, *p* < 0.01; ***, *p* < 0.001). **(D)** Mean rcn of *tax* and the Tax-target genes *4-1BB* and *HIAP-1*, normalized to the housekeeping gene *β-Actin*, were correlated and subjected to Pearson correlation analysis (Pearson correlation coefficient r = 0.95 and r = 0.84 respectively, *p* = 1.11E-06 and *p* = 5.37E-04 respectively).

Next, the chronically HTLV-1-infected T-cell line MT-2 (28) was employed to investigate the influence of the HDACi treatment on the integrated HTLV-1 provirus. This contrasts with previous experiments, which relied on transient transfection of the LTR-Tax plasmid in Jurkat T-cells. MT-2 cells were incubated with the indicated concentrations of Panobinostat, Belinostat, and Romidepsin for 24 h. Subsequently, flow cytometry staining with a LIVE/DEAD stain and an Annexin V conjugate was performed to determine the proportion of viable, early apoptotic, and late apoptotic and necrotic cells. 100 nM and 500 nM Panobinostat and 50 nM Romidepsin significantly reduced the frequency of viable cells (**Figure 4A**). 500 nM Panobinostat also significantly enhanced the amount of late apoptotic and necrotic cells. Especially, Panobinostat and Romidepsin reduced the viability of the chronically infected cells dose-dependently, whereas Belinostat had little influence on cell vitality. These findings agree with other studies that found that 25-50 nM Panobinostat strongly induces apoptosis in MT-2 cells and other ATL-related cell lines (47) and that 3.6 nM Romidepsin (calculated with molecular weight 540.7 g/mol) amplifies Annexin V positivity significantly in MT-2 cells (51).

### Selected HDACi dose-dependently enhance *tax* transcription, but not Tax protein expression in chronically infected cells

Next, we examined the influence of the HDACi Panobinostat, Belinostat, and Romidepsin on Tax protein expression and transcription in MT-2 cells by intracellular Tax staining followed by flow cytometry (**Supplementary Figure S4A**) and qRT-PCR, respectively. Apart from the positive control treatment with PMA+Ionomycin (110%), only 10 nM Panobinostat (133%) and 10 nM Romidepsin (134%) produced a significant intensification of Tax protein above the solvent control level of DMSO (dotted line) (**Figure 4B**). Considering the impact on the percentage of Tax-expressing cells led to similar results (**Supplementary Figure S4B**). In this case, only 10 nM and 100 nM Panobinostat (136% and 116% respectively) and PMA+Ionomycin (109%) treatment significantly raised Tax frequency above the solvent control. Observing the level of Tax protein expression in the MT-2 cells, there seemed to be no dose-dependent impact of the HDACi administration discernible. Previous studies revealed that 5 ng/ml Romidepsin (≈ 9.2 nM, calculated with molecular weight 540.7 g/mol) increases Tax protein expression in MT-2 cells (51), and both 20 nM and 200 nM Panobinostat boosted Tax expression in MT1GFP cells (50). In agreement with the present results, no dose-dependency was observed in the treatment of MT1GFP cells with the two concentrations of Panobinostat.

qRT-PCR was employed to evaluate the effect of the HDACi administration on *tax* transcription. Primarily, Panobinostat and Romidepsin could substantially and dose-dependently raise *tax* mRNA levels (**Figure 4C**). In contrast, Belinostat had no significant impact on *tax* transcription. The superiority of Panobinostat and Romidepsin to Belinostat was reproducible on the Tax-target gene level, represented by *4-1BB* and *HIAP-1* quantification (**Supplementary Figure S3B**, upper and middle panel). *tax* mRNA correlated very well and significantly with its target genes (**Figure 4D**). By contrast, although the non-Tax-target gene *FOXP3* also significantly increased upon HDACi treatment, expression of *FOXP3* remained ca. two logarithmic orders lower than expression of the target genes (**Supplementary Figure S3B**, lower panel (54)). These findings on the transcriptional level in MT-2 cells (**Figure 4C, D**; **Supplementary Figure S3B**) paralleled the results from the transiently transfected Jurkat T-cells (**Figure 3C, D**; **Supplementary Figure S2B**).

### Toxicity of Panobinostat and Romidepsin on lymphocytes from HTLV-1 infected patients is time-dependent

We then evaluated further how the HDACi treatment affects the spontaneous burst of Tax expression in *ex vivo* cells from HTLV-1 infected patients. It has been established that plus-strand expression of HTLV-1 is usually undetectable in freshly isolated PBMCs. Thereafter, Tax expression increases strongly and spontaneously upon *ex vivo* culture, reaching a maximum after six to twelve hours of culture, after that decreasing again (7, 55, 56). We sought to modify this spontaneous burst, which is currently the best available *in vitro* model for latent HTLV-1 infection. We used samples from five HTLV-1 infected individuals who were either asymptomatic carriers or had HAM/TSP (**Table 2**) and all had a proviral load of >1 HTLV-1 DNA copy per 100 PBMCs. CD4^+^ T-cells represent the main latent reservoir of HTLV-1 infection and were therefore isolated by negative selection prior to culture. Removal of CD8^+^ T-cells prevented CTL-mediated killing of the infected cells during the culture. Since Panobinostat and Romidepsin were most potent in inducing Tax expression in MT-2 cells (above), one concentration of each HDACi was selected for further experiments in primary cells. 50 nM Panobinostat and 5 nM Romidepsin were initially administered on d0. On d2 of culture, the cells received a second dose, equivalent to a quarter of the initial treatment on d0. Samples were harvested on d0, d1, and d4.

**Table 2:** Patient PBMC samples used for ex vivo analysis.

Patient	Disease	PVL
P1	AC	4.60
P2	AC	2.60
P3	HAM	11.60
P4	AC	8.40
P5	AC	4.30

AC, asymptomatic carrier; HAM, HTLV-1-associated myelopathy/tropical spastic paraparesis; PVL, proviral load is the number of HTLV-1 DNA copies per 100 PBMCs.

**Figure 5:**
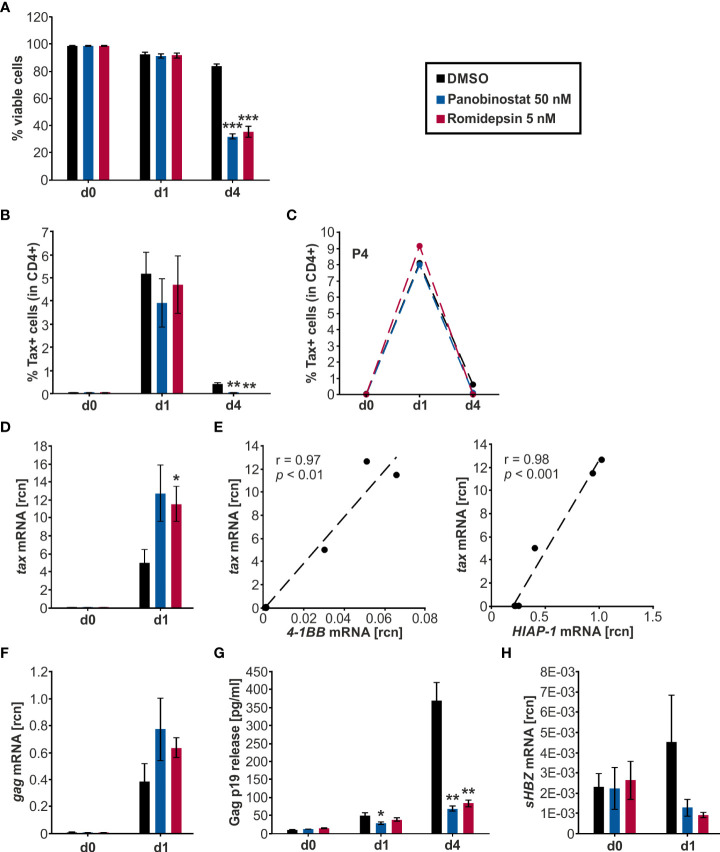
Panobinostat and Romidepsin enhance *tax* transcripts but inconsistently Tax protein. **(A–H)** CD4^+^ T-cells isolated from five HTLV-1 infected patients were treated with Panobinostat (50 nM; blue), Romidepsin (5 nM; pink), or the solvent control DMSO (black) on d0. The cultured cells received a second treatment on d2, equivalent to a quarter of the initial treatment of d0. Cells were analyzed *via* **(A–C)** flow cytometry, **(D–F, H)** qRT-PCR, and **(G)** ELISA, on days 0, 1, and 4. **(A)** Cell viability was determined by flow cytometry based on LIVE/DEAD staining. Mean values of the five patient samples (P1-P5) ± SE are depicted, and values were compared to the respective solvent-treated cells (DMSO) using Student’s t-test (***p < 0.001). **(B)** Intracellular Tax expression was analyzed *via* flow cytometry using the LIVE/DEAD Near-IR Dead Cell Stain Kit, CD4, and Tax (Lt-4 mAB) labeling (gating strategy: **Supplementary Figure 5A**). The mean percentage of intracellular Tax protein expression of the five patient samples ± SE was compared to DMSO treatment using Student’s t-test (**p < 0.01). **(C)** The individual frequency of Tax expression of patient 4 (P4) is plotted. **(D)** Mean rcn of *tax*, normalized to those of the housekeeping gene *β-Actin*, of the five patients ± SE were compared using Student’s t-test (*p < 0.05 relative to DMSO). **(E)** Mean rcn of *tax* and Tax-target genes *4-1BB* and *HIAP-1*, normalized to the housekeeping gene *β-Actin*, were correlated and subjected to Pearson correlation analysis (Pearson correlation coefficient r = 0.97, and r = 0.98 respectively, *p* = 1.18E-03, and *p* = 2.43E-04 respectively). **(F)** Mean rcn of g*ag*, normalized to those of the housekeeping gene *β-Actin*, of the five patients ± SE were compared using Student’s t-test (all *p* > 0.05 relative to DMSO). **(G)** The release of Gag p19 was assessed *via* ELISA. The mean of the five patient samples of the absolute Gag p19 concentration ± SE is represented and was compared using Student’s t-test (*p < 0.05; **p < 0.01, relative to DMSO). **(H)** Mean rcn of *sHBZ*, normalized to those of the housekeeping gene *β-Actin*, of the five patients ± SE were compared using Student’s t-test (all *p* > 0.05 relative to DMSO).

Flow cytometry was employed to quantify the viability of the cells throughout the treatment period (**Figure 5A**). Samples started out with 98-99% viable cells and maintained a 91-92% viability on d1, with no significant difference between the HDACi treatment and the solvent control DMSO. During the subsequent culture period, the viability of the DMSO-treated cells dropped significantly to 84 % on d4 compared to d1. However, the HDACi treatment significantly impaired cell viability compared to DMSO on d4. Panobinostat and Romidepsin resulted in 32% and 36% viable cells, respectively. These results are in agreement with earlier findings showing that the HDACi Valproate was also proapoptotic in PBMCs from HTLV-1-infected patients and lymphocytes from healthy patients (20, 21).

### HDACi consistently raise *tax* and *gag* transcription *ex vivo* but display patient-specific kinetics in Tax protein expression in lymphocytes from HTLV-1 infected patients

To assess the impact of Panobinostat and Romidepsin treatment on Tax protein expression, intracellular Tax was stained in CD4^+^ T-cells on d0, d1, and d4 of culture with either 50 nM Panobinostat, 5 nM Romidepsin, or the solvent control DMSO. At the beginning of the culture period, barely any Tax protein expression (< 0.4%) was detectable (**Figure 5B**). During the subsequent treatment period, the spontaneous burst in Tax protein was clearly visible on d1 (**Figure 5B**, **Supplementary Figure S5C**). Mean percentages of Tax expression in five patients rose to 5.2% in DMSO-treated, 3.9% in Panobinostat, and 4.7% in Romidepsin treated CD4^+^ T-cells (**Figure 5B**). After that, Tax protein levels declined until d4. Overall, the HDACi treatment did not significantly enhance Tax frequency and intensity on d1 and d4 (**Figure 5B**, **Supplementary Figure S5C**). However, the d4 Tax frequency was significantly lower in HDACi treated cells than in DMSO (**Figure 5B**). Examining the individual data from the five patients highlights that there were patient-specific differences in the kinetics of Tax expression. In patient four (P4; **Figure 5C**) and patient one (P1; **Supplementary Figure S5D**, left panel), the Romidepsin administration caused an increase in Tax frequency compared to the solvent control on d1 of the time course. However, in three out of five patients, HDACi treatment caused a decrease in Tax protein expression (**Supplementary Figure S5D**). In summary, the measurement of Tax protein yielded inhomogeneous results between the patients. Tax expression under treatment with Romidepsin was consistently higher than Tax protein levels under Panobinostat treatment. Results from the quantification of *tax* transcription stood in contrast to the inconsistent Tax protein expression. Both Panobinostat and Romidepsin treatment enhanced *tax* transcription in all five patients on d1 of *ex vivo* culture (**Figure 5D**). This uniform boost was significant for Romidepsin compared to DMSO. Analogous to the transiently transfected Jurkat T-cells (**Figures 2B-D**, **3B,C**) and the chronically infected MT-2 cells (**Figures 4C, D**), the increase in *tax* transcription also translated to the Tax-target gene level, resulting in a very good and significant correlation with *4-1BB* and *HIAP-1* mRNA levels (**Figure 5E**; **Supplementary Figure S5E**, left and middle panel). Conversely to the Tax-target genes, the non-Tax-target gene *FOXP3* was repressed by the HDACi on d1 (**Supplementary Figure S5E**, right panel). Quantification of transcripts was impossible on d4 due to lacking sample amount.

To investigate whether the findings concerning *tax* transcription and Tax protein expression in the HTLV-1 infected CD4^+^ T-cells also apply to other genes of HTLV-1, we evaluated Gag expression. *Gag* is a sense-strand-encoded structural gene present in all replication-competent retroviruses (57). *Gag* mRNA was boosted by the Panobinostat and the Romidepsin treatment, albeit not significantly (**Figure 5F**). In addition, *gag* expression rose concomitantly with *tax* mRNA, and transcription of those two sense strand genes correlated very well (Pearson correlation r = 0.99; *p* = 8.995E-05; data not shown). Gag protein release, together with the HTLV-1 core protein p19 into the culture supernatant was quantified by ELISA (**Figure 5G**). The amount of Gag in the supernatant rose during the culture period, due to the continuous release. However, this release of Gag was impaired in the samples treated with Panobinostat or Romidepsin compared to DMSO. This inhibition was already visible on d1 and even more prominent on d4. Panobinostat impaired the release significantly on d1 and d4 and Romidepsin on d4. The repression occurred in all patients, except for the Romidepsin treatment in P2 on d1. Collectively, these findings parallel the expression of Tax in the aspect that an induced transcription was not adequately translated into protein. Deteriorating cell viability can only partially explain the diminished Gag release.

These results show that HDACi treatment robustly increases transcription of the sense-strand of HTLV-1. In two out of five patients, the induction of *tax* mRNA resulted in a slight induction of Tax protein; in three out of five, the rise in *tax* mRNA had not impact on the protein level. These findings suggest patient-specific obstacles between plus-strand transcription and translation, potentially due to differences in reservoir size, genetic background, gender, or type of therapy, as observed for HIV (58).

### Anti-sense transcription of *sHBZ* is regulated inversely to sense strand transcription

Next, we examined the effects of Panobinostat and Romidepsin on HBZ transcription *via* qRT-PCR (**Figure 5H**). *HBZ* served as a surrogate for the minus-strand transcription of HTLV-1. In contrast to Tax, which is only expressed in infrequent bursts, HBZ is continuously produced, albeit at a very low level (59, 60). The *HBZ* gene exists as unspliced (*usHBZ*) and spliced version (*sHBZ*), whereas the sHBZ protein inhibits Tax-mediated transcriptional activation of the sense strand more strongly than usHBZ (38).Thus, we decided to focus on *sHBZ*. The level of detectable *sHBZ* was in general very low, compared to the other quantified viral genes *tax* and *gag*. On d1, *sHBZ* mRNA levels were lower in the cells treated with Panobinostat or Romidepsin than in the solvent control DMSO. However, this observed impact was not significant (**Figure 5H**).

Previous experiments with PBMCs from HAM/TSP patients demonstrated that while Valproate activates plus-strand transcription, HBZ expression from the minus-strand was inhibited (20). Thus, our data emphasize that this differing effect on sense- and anti-sense transcription is not specific for the HDACi Valproate but also displayed by the HDACi Panobinostat and Romidepsin.

## Discussion

### Key findings with Panobinostat and Romidepsin point towards a post-transcriptional block, which was not observed in Valproate trials

Attempts to boost Tax expression in latently infected T-cells of people living with HTLV-1, to expose those cells to the host’s immune response, primarily focused on the HDACi Valproate. Despite a transient decrease in the PVL of HAM/TSP patients, Valproate treatment failed to achieve a permanent reduction (20, 21, 25). We hypothesized that more potent or more selective HDACi might prove superior to Valproate in manipulating Tax expression. In this study, we demonstrated that Panobinostat, Belinostat, and Romidepsin resulted in a higher rate of *tax* transcription than Valproate in transiently transfected Jurkat T-cells. Therefore, these three HDACi were selected for further dose response experiments in transiently transfected Jurkat T-cells and chronically infected MT-2 cells. Panobinostat and Romidepsin proved to be the most successful at enhancing especially *tax* transcription, but less so Tax protein expression. In consecutive experiments in CD4^+^ T-cells from people living with HTLV-1, the HDACi robustly induced sense transcription, including *tax* and *gag* genes. *Tax* transcription also translated to the Tax-target gene level in all of the model systems, resulting in enhanced *4-1BB* and *HIAP-1* expression. Unlike the raised sense transcription, the anti-sense transcription of *sHBZ* was repressed. Interestingly, there were patient-specific kinetics present in the translation to protein. Induction of *tax* mRNA only resulted in increased Tax protein in two out of five patients. The boost in *gag* transcription did not raise Gag release, but rather Gag release was repressed by the HDACi treatment with Panobinostat and Romidepsin. The discrepancy between raised *tax*, Tax-target, and *gag* transcription and only inconsistently enhanced protein expression pointed towards a post-transcriptional block in the sense-strand expression of HTLV-1.

This raises the question why this putative post-transcriptional block was not witnessed in previous studies investigating the HDACi Valproate. It has been demonstrated that 2 mM Valproate enhances the release of HTLV-1 p19 core protein from PBMCs from HAM/TSP patients over 48 h. In an explorative trial with four HAM/TSP patients, Valproate reached a serum concentration from 0.31-0.65 mM (calculated from 45 to 93 µg/ml with molecular weight 143.2 g/mol). In these patients, the HDACi treatment transiently increased the PVL at first, but thereafter the PVL decreased compared to the initial PVL (21). However, a two-year trial of HAM/TSP patients with Valproate showed that significant long-term suppression of the PVL is not achievable (25). Another *ex vivo* study found that 5 mM Valproate boosts *tax* transcription and Tax protein expression in CD4^+^ T-cells from people living with HTLV-1. The boosted *tax* transcription, induced by 5 mM Valproate, peaked at a high level on d1 and declined over the remaining *ex vivo* culture period of five days. Furthermore, the increase in Tax protein compared to nontreated samples was significant on d1 of culture. Thereafter, on d2, there was a decrease in Tax protein observable. Release of the viral protein Gag was also stimulated by 5 mM Valproate after 2d of cell culture. This protein induction was concomitant with a rise in g*ag* mRNA (20). By contrast, in our study Panobinostat and Romidepsin did not induce Tax protein expression as robustly as Valproate. This might be due to the selected dosing. The reported results were obtained with 5 mM Valproate, which modified HTLV-1 gene expression profoundly. This fundamental alteration of HTLV-1 gene expression together with possible off-target effects, might “override” presumed post-transcriptional blocks and result in a significant induction of Tax protein expression *ex vivo*. Yet, it is crucial to note that conditions evoked by 5 mM Valproate under cell culture circumstances are not feasible *in vivo*. Other studies describe achievable plasma concentrations of Valproate to be 0.25-0.6 mM or 1-2 mM (25, 61). In patients treated for HIV-1 latency reversal, Panobinostat reaches 50-60 nM peak plasma concentrations in its lowest dosing schedule (62). Adequate plasma concentrations of Romidepsin are 128-247 nM (reported as 69 ng/ml to 134 ng/ml, calculated with molecular weight 540.7 g/mol) (63). Therefore, our employed concentrations of 50 nM Panobinostat and 5 nM Romidepsin chosen in our *ex vivo* study of lymphocytes from people living with HTLV-1 mirror *in vivo* conditions more appropriately.

Furthermore, Valproate is a short-chain fatty acid HDACi, and this group of HDACi is known for its poor bioavailability and low HDAC inhibitory activity (18, 19). It is essential to consider that besides HDAC inhibition, Valproate has several other pharmacodynamic targets. These include induction of GABA signalling, inhibition of sodium channels, increase in glutamate signalling, modulation of DNA methylation, and indeed activation of the MAPK/ERK pathway (64, 65). Recently, this pathway has been shown to be implicated in the induction of sense-strand transcription of HTLV-1. Cellular stress, e.g., hypoxia, is sensed by p38-MAPK, and activation of p38-MAPK leads to increased *tax* transcription (66). This off-target effect might also contribute to Valproate being able to reactivate HTLV-1 from latency.

Still, it is surprising that the rise in *tax* mRNA expression on d1, attributed to Panobinostat and Romidepsin application, was not translated into a consistent rise in Tax protein. A previous study of PBMCs from four HAM/TSP patients found that *tax* mRNA and Tax protein expression correlate closely during the first 48 h of *ex vivo* culture (67). Yet, this correlation was not observed once the cells were treated with the HDACi Panobinostat and Romidepsin in our study. In contrast to Valproate, the mode of action of the cyclic peptide HDACi Romidepsin is presumably more limited to epigenetic modification. Interestingly, a trial with HIV-1 infected patients showed that Romidepsin preferentially increases early events in HIV transcription (initiation and elongation) (68). Consequently, the HDACi treatment only relieved a portion of the post-transcriptional blocks. However, since certain HDACi induce autophagy in T-cells (69), this might also explain the discrepancy between increased *tax* mRNA and only moderately enhanced Tax protein levels, which has to be addressed in future studies. Further agents are required to alleviate other barriers (completion of transcription, multiple splicing, translation) to retroviral reactivation in combination with HDACi treatment. Furthermore, retroviral gene expression cannot be looked at in isolation since one decisive determinant of transcription is the proviral integration site in the host genome. There are typically 10^4^ to 10^5^ individual clones found in one HTLV-1 infected person (4, 7). An HIV-1 barcoding technology demonstrated that different LRAs, phytohemagglutinin, and the HDACi Vorinostat, reactivated proviruses inserted at distinct genomic locations (70, 71). This is a critical step in the direction of understanding why some latently infected cells can be induced and others not.

Retroviral latency is tightly regulated, and there are several diverse barriers to a comprehensive reactivation from latency. In the related retrovirus HIV-1, it has been described that large quantities of the latent reservoir could be reactivated in the course of kick-and-kill trials, but elimination of those was ultimately insufficient (12). *In vitro* data using a primary CD4^+^ T-cell model also documented that HDACi treatment with Vorinostat increased viral transcription but only limited viral translation (52). These findings with HIV-1 parallel the results from our investigation of Panobinostat and Romidepsin in HTLV-1 infected CD4^+^ T-cells. These characteristics of HDACi treatment might be attributed to HDACi only targeting one facet of the transcriptional and post-transcriptional blocks implicated in latency. Comprehensively, they comprise epigenetic blocks, transcription initiation blocks, transcription elongation blocks, and post-transcriptional blocks (58). One example of a post-transcriptional block is the downregulation of Matrin 3 (MATR3) in latently-infected patients’ cells. The nuclear matrix protein MATR3 is a positive regulator and post-transcriptional cofactor of HIV-1. Ectopic overexpression of MATR3 rescued HIV-1 reactivation under HDACi treatment (72).

Since Tax induction by the HDACi treatment also profoundly alters cellular gene expression, it is essential to consider these effects. Tax expression has been associated with both increased cell death and protection from apoptosis (50, 73, 74). A caveat of the kick-and-kill approach is that protection from apoptosis might render the infected cells less responsive to a cytotoxic T-cell response. An intriguing method to target this is *via* inhibition of HIF-1α. This might circumvent cell death resistance in infected cells and provides a further direction of research (73).

Our study demonstrated that the HDACi Panobinostat and Romidepsin robustly activate *tax* transcription in all examined model systems, but only moderately impacted Tax protein levels. This calls into question whether HDACi have a sufficiently strong potential to reactivate HTLV-1, to justify their use in the kick-and-kill approach. We argue that due to the complex nature of retroviral latency a multi-facetted approach will be vital to effectively expose latently infected cells to the host’s immune response. Therefore, a combination treatment that addresses multiple aspects of blocks in latency reversal possesses the greatest promise. Our data highlights that HDACi can be a key component of the kick-and-kill approach.

### HDAC- and HDACi-specific implications

Only little is known about the HDAC complex formation at the HTLV-1 LTRs, besides the fact that it differs between the 5’LTR and 3’LTR. HDAC 1 and 2 favor the 5’LTR, and HDAC 3 preferentially binds at the 3’LTR (26). These are all class I HDACs (13). It is questionable how the selectivity of the HDACi might influence HTLV-1 reactivation since so little is known about the distribution of other HDACs in HTLV-1 infected lymphocytes.

Panobinostat is a pan-HDACi, and Romidepsin selectively inhibits class I HDACs (13). Despite their differential selectivity, side effect profiles of Romidepsin and Panobinostat seem comparable. However, a study found that Romidepsin is disproportionally toxic to activated T-cells, especially CTLs (75). In our study, especially Panobinostat and Romidepsin induced cell death. However, generally it is not surprising that HDACi treatment induces apoptosis, considering that HDACi are commonly also used as anticancer agents due to their proapoptotic potential (13).

Furthermore, it is crucial to consider, that HDACi with the same selectivity and members of the same HDACi class can still display different potency. This applies to Panobinostat and Belinostat in our study, both hydroxamate pan-HDACi. Panobinostat proved to be superior to Belinostat in transiently transfected Jurkat T-cells and chronically infected MT-2 cells in inducing *tax* transcription. Belinostat’s inferiority in inducing Tax expression aligns with it having very little influence on cell vitality. Panobinostat being more promising than Belinostat in the context of HTLV-1 latency reversal is consistent with the finding that Panobinostat was also more potent than Belinostat in inducing production of the related retrovirus HIV in latently infected primary cells (61).

Importantly, Tax can also translocate to the cytoplasm and interact with HDAC 6 there. This interaction inhibits the formation of stress granules, which would result in sequestration and silencing of mRNA. Thereby, Tax allows the efficient translation of proteins even during cellular stress (76). HDAC 6 is a member of the class IIb HDACs (13). Thus, it is not impaired by Valproate or Romidepsin but by Panobinostat. Therefore, Panobinostat, as pan-HDACi, may possess additional potential in inducing HTLV-1 reactivation on multiple levels.

Additionally, the killing of infected cells through CTLs might be affected by HDACi treatment. On the one hand, concerns focus on the lytic efficiency of CTL cells (12). However, there is evidence that at least Valproate does not impair the lytic efficiency of CTL cells in HTLV-1 infected patients (25). On the other hand, recent data submitted for publication showcased that *ex vivo* Panobinostat treatment drastically downregulated the expression of interferon-regulated genes in lymphocytes from an HIV-1 infected patient, potentially jeopardizing the killing (77). Therefore, further investigations should evaluate whether this alteration in gene expression is also of consequence in HTLV-1 infection and how efficient killing of infected cells can be ensured.

### Contribution and conclusion

While it might not currently be feasible to reactivate all HTLV-1-infected cells, this might still be a worthwhile endeavor. This idea is supported by the fact that the PVL of a patient can serve as a biological marker and risk factor for the development of ATL and HAM/TSP and the worsening of HAM/TSP (78–81). The PVL of a patient is proportional to the amount of distinct infected T-cell clones. A higher oligoclonality is associated with ATL than with HTLV-1 infected individuals without this malignant condition. The number of unique integration sites is higher in patients with HAM/TSP than in asymptomatic carriers of HTLV-1 (82). Thus, even if the treatment with HDACi as latency-reversing agents can only reactivate a portion of latently infected clones, and these can consequently be eradicated, this might already lower the risk of developing ATL and HAM/TSP, or prevent deterioration of the clinical state.

This study provides evidence for the potential of Panobinostat and Romidepsin to be further investigated, perhaps in combination with other compounds, to reactivate HTLV-1 from latency. Our results highlight that these HDACi can contribute to relieving an epigenetic block in HTLV-1 latency. Further studies will have to determine which drug combinations can best reactivate different subsets with distinct integration sites from latency and relieve additional blocks.

## Data availability statement

The original contributions presented in the study are included in the article/**Supplementary Material**. Further inquiries can be directed to the corresponding author.

## Ethics statement

The studies involving human participants were reviewed and approved by National Research Ethics Service, London, UK. The patients/participants provided their written informed consent to participate in this study.

## Author contributions

AS performed most experiments, analyzed the data, and wrote the manuscript. SK performed experiments. AA helped with the analysis of patient samples. GT and CB provided the patient samples and assisted in designing and interpreting experiments. AT-K conceived and supervised the project, analyzed the data, and wrote the manuscript. All authors contributed to the article and approved the submitted version.

## Funding

This research was funded by Deutsche Forschungsgemeinschaft (DFG), GRK2504, project number 401821119, subproject A2 (to AT-K). AT-K was further supported by the DFG grant TH2166/1-1 and 1-2, by the BMBF (Milk-TV, 01KI2023), and by the Interdisciplinary Center for Clinical Research (IZKF) at the Medical Faculty of Friedrich-Alexander-Universität Erlangen-Nürnberg (FAU) (Project A91). AS was supported by the “Sofie Wallner Preis für Krebsforschung” from the “Sofie Wallner Stiftung” at FAU.

## Acknowledgments

We thank Sebastian Millen for designing the tax forward primer and for helpful discussions. We thank Aileen Rowan (Imperial College, London) for helpful discussions. We thank Dominic Smith, Danay Koftori (both Imperial College London), Stefanie Heym, and Norbert Donhauser (both Universitätsklinikum Erlangen) for practical assistance. We are grateful to Stefan Koch, Linköping University, Linköping, Sweden, for providing FLAG-FOXP3 (Addgene plasmid #153147). We acknowledge support by Deutsche Forschungsgemeinschaft and Friedrich-Alexander-Universität Erlangen-Nürnberg (FAU) within the funding program Open Access Publication Funding. The present work was performed in fulfillment of the requirements for obtaining the degree “Dr. med.” (for AS).

## Conflict of interest

The authors declare that the research was conducted in the absence of any commercial or financial relationships that could be construed as a potential conflict of interest.

## Publisher’s note

All claims expressed in this article are solely those of the authors and do not necessarily represent those of their affiliated organizations, or those of the publisher, the editors and the reviewers. Any product that may be evaluated in this article, or claim that may be made by its manufacturer, is not guaranteed or endorsed by the publisher.
