# Supplementary material for: HDAC inhibitors Panobinostat and Romidepsin enhance tax transcription in HTLV-1-infected cell lines and freshly isolated patients’ T-cells
Source: Front Immunol. 2022 Aug 16;13:978800. doi: 10.3389/fimmu.2022.978800 (PMC9424546; doi:10.3389/fimmu.2022.978800)
Supplement: Supplementary file 1 [file DataSheet_1.pdf]

Supplementary Material

1 Supplementary Figures

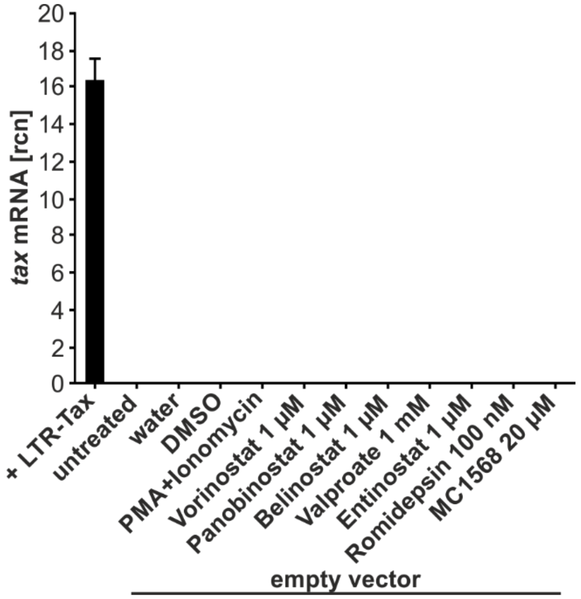

Supplementary Figure 1. Detection of *tax* mRNA by qRT-PCR. Jurkat T-cells were transiently transfected with empty vector DNA pEF1α or 15 µg of the plasmid LTR-Tax (and 10 µg of pGL3-U3R-Luc and 25 µg of empty vector DNA pcDNA3.1). At 24 h after transfection, chemical treatment was carried out for 24 h. Mean relative copy numbers (rcn) of *tax*, normalized to the house-keeping gene *β-Actin*, of three independent experiments ± standard error (SE) are depicted. The sample transfected with LTR-Tax was measured as technical triplicate and served as a positive control.

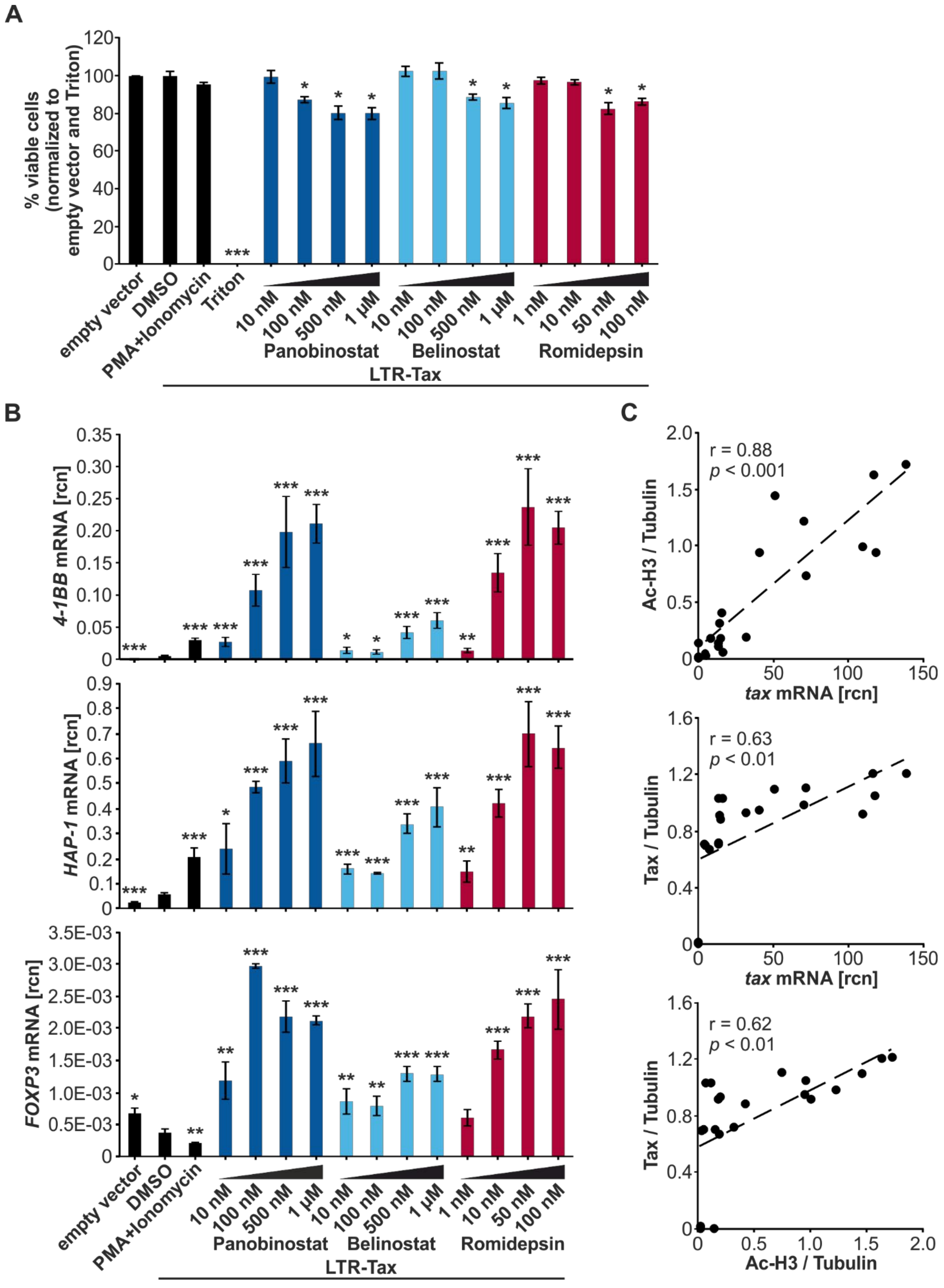
A
% viable cells (normalized to empty vector and Triton)
empty vector
DMSO
PMA+Ionomycin
Triton
10 nM
100 nM
500 nM
1 µM
1 nM
50 nM
100 nM
Panobinostat
Belinostat
Romidepsin
LTR-Tax
B
4-1BB mRNA [rcn]
HAP-1 mRNA [rcn]
FOXP3 mRNA [rcn]
C
r = 0.88
p < 0.001
Ac-H3 / Tubulin
tax mRNA [rcn]
r = 0.63
p < 0.01
Tax / Tubulin
r = 0.62
p < 0.01

Supplementary Figure 2. Apoptosis detection and qRT-PCR in Jurkat T-cells. (A, B, C) Jurkat T-cells were transiently transfected with 15 µg LTR-Tax and filled with the empty vector DNA pEF1α to 50 µg. Increasing doses of the HDACi Panobinostat or Belinostat (10 nM, 100nM, 500 nM, 1 µM), or Romidepsin (1 nM, 10 nM, 50 nM, 100 nM) were added at 24 h post transfection for 24 h. PMA+Ionomycin served as a positive control for activation of transcription and dimethyl sulfoxide (DMSO) as solvent control. **(A)** A lactate dehydrogenase (LDH) release assay was performed after 24 h of treatment. Cells treated with Triton (dead cells) and empty vector transfected cells were set as 0 % and 100 % viable cells, respectively. Mean values of three independent experiments ± SE are depicted and were compared using Student's t-test (*, $p < 0.05$; **, $p < 0.01$; ***, $p < 0.001$ relative to DMSO). **(B)** Mean relative copy numbers (rcn) of *4-1BB*, *HIAP-1*, and *FOXP3* normalized to the house-keeping gene *β-Actin*, of three independent experiments ± SE were compared to the DMSO-treated cells using Student's t-test (*, $p < 0.05$; **, $p < 0.01$; ***, $p < 0.001$). **(C)** Western Blot analysis and subsequent densitometric quantification of Tax protein, acetylated Histone H3 (Ac-H3), and α-Tubulin (Tubulin) as the loading control was performed. Mean rcn of *tax*, normalized to the house-keeping gene *β-Actin*, and Ac-H3 / Tubulin protein were correlated and subjected to Pearson correlation analysis (Pearson correlation coefficient r = 0.88; p = 1.4E-07). Pearson correlation analysis was also carried out for mean rcn of *tax*, normalized to *β-Actin*, and Tax / Tubulin (Pearson correlation coefficient r = 0.63; p = 2.1E-03), and for Ac-H3 / Tubulin, and Tax / Tubulin (Pearson correlation coefficient r = 0.62; p = 2.7E-03).

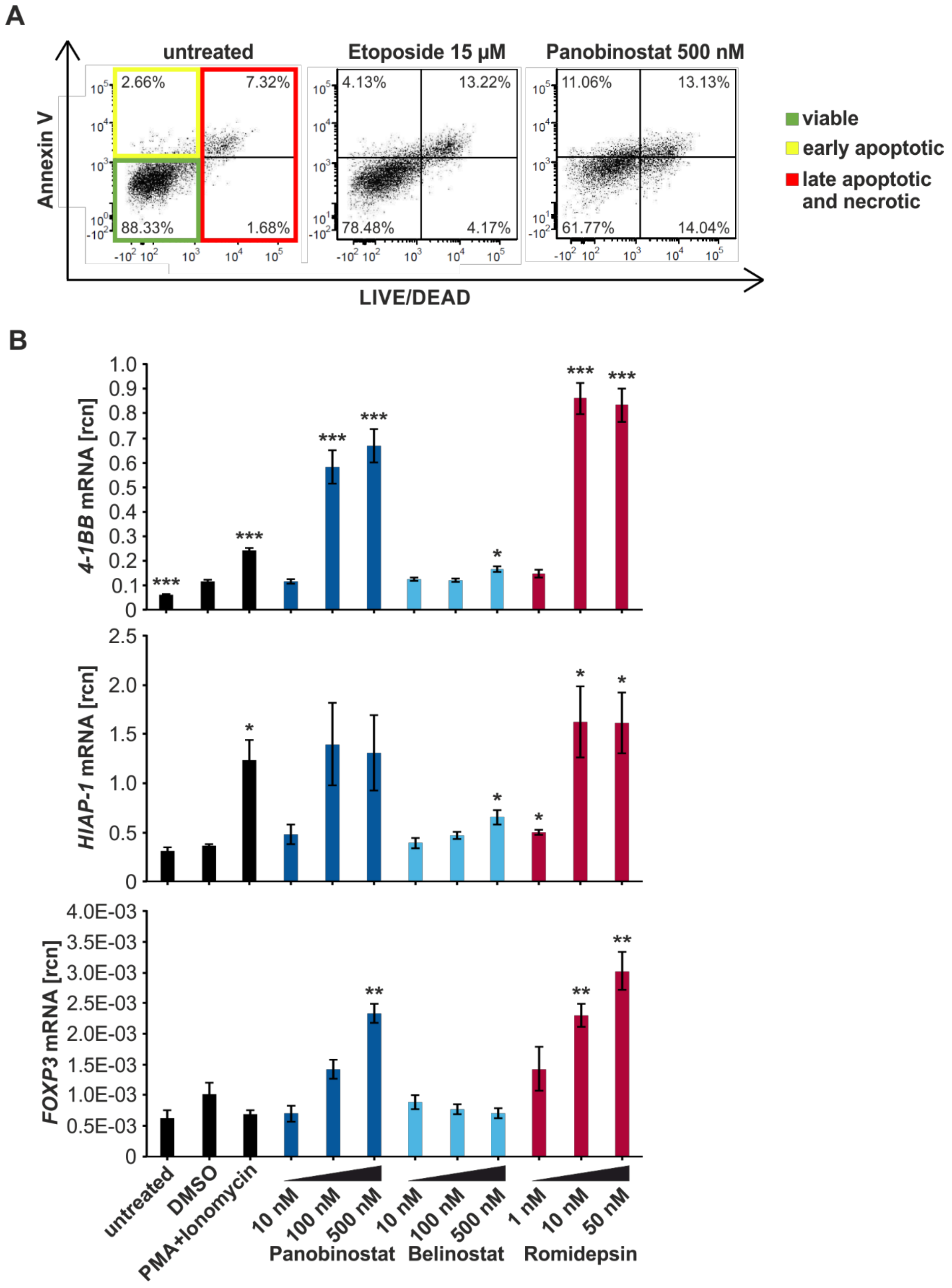
A
untreated
Etoposide 15 µM
Panobinostat 500 nM
Annexin V
LIVE/DEAD
2.66%
7.32%
88.33%
1.68%
4.13%
13.22%
78.48%
4.17%
11.06%
13.13%
61.77%
14.04%
viable
early apoptotic
late apoptotic and necrotic
B
4-1BB mRNA [rcn]
HIAP-1 mRNA [rcn]
FOXP3 mRNA [rcn]
untreated
DMSO
PMA+Ionomycin
10 nM
100 nM
500 nM
Panobinostat
10 nM
100 nM
500 nM
Belinostat
1 nM
10 nM
50 nM
Romidepsin

Supplementary Figure 3. Apoptosis detection and qRT-PCR in MT-2 cells. (A, B) MT-2 cells were treated with increasing concentrations of the HDACi Panobinostat, Belinostat, or Romidepsin as indicated for 24 h. PMA+Ionomycin served as a positive control for activation of latent viral transcription and DMSO as solvent control. **(A)** Gating strategy upon staining cells with the LIVE/DEAD Fixable Far Red Dead Cell Stain Kit and an Annexin V pacific blue conjugate is displayed. Etoposide (15 µM) served as a positive control for induction of apoptosis. The frequencies of viable cells (double-negative cells; green), early apoptotic cells (LIVE/DEAD$^-$/Annexin V$^+$; yellow), or late apoptotic and necrotic cells (LIVE/DEAD$^+$; red) were quantified by flow cytometry. **(B)** Mean relative copy numbers (rcn) of *4-1BB*, *HIAP-1*, and *FOXP3* normalized to the house-keeping gene *β-Actin*, of four independent experiments ± SE were compared to the DMSO-treated cells using Student's t-test (*, $p < 0.05$; **, $p < 0.01$; ***, $p < 0.001$).

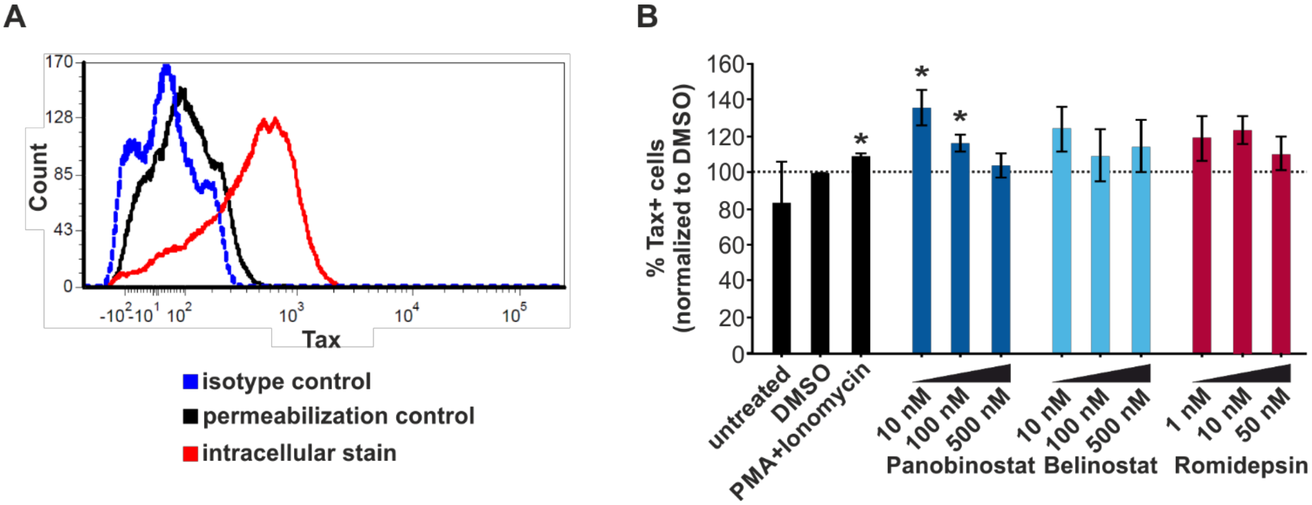

Supplementary Figure 4. Tax expression in MT-2 cells. (A, B) MT-2 cells were treated with increasing concentrations of the HDACi Panobinostat, Belinostat, or Romidepsin as indicated for 24 h. PMA+Ionomycin served as a positive control for activation of latent viral transcription, DMSO as solvent control. Flow cytometry was performed to assess Tax protein in living cells. **(A)** Expression of intracellular Tax protein compared to the permeabilization (black) and isotype (blue) control in DMSO treated cells. **(B)** Percentage of Tax expressing (Tax+) cells, normalized to DMSO, of three independent experiments ± SE are depicted, and values were compared to DMSO treatment using Student's t-test (*, $p < 0.05$).

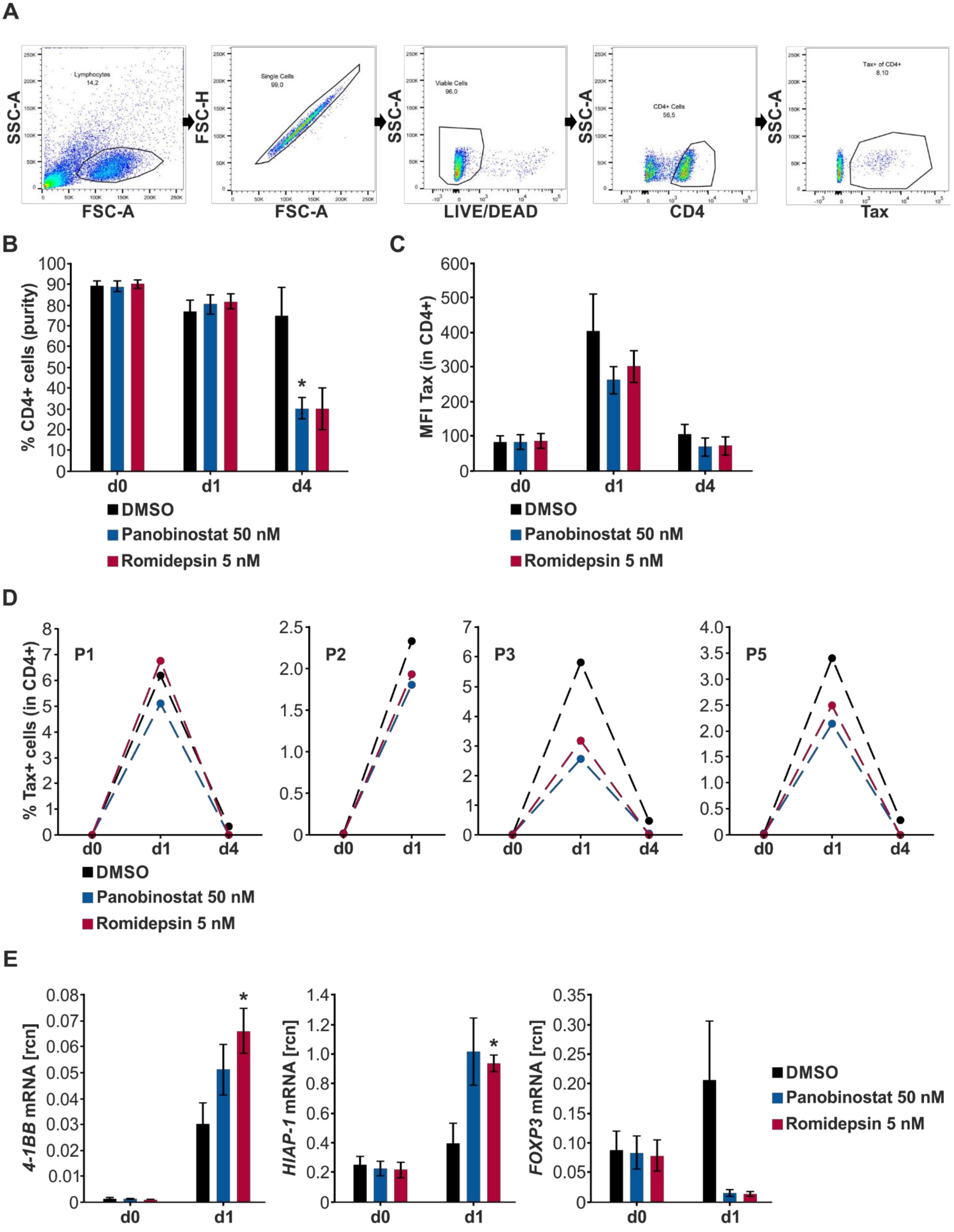
A
Lymphocytes
14,2
SSC-A
FSC-A
Single Cells
99,0
FSC-H
FSC-A
Viable Cells
96,0
SSC-A
LIVE/DEAD
CD4+ Cells
56,5
SSC-A
CD4
Tax+ of CD4+
8,10
SSC-A
Tax
B
% CD4+ cells (purity)
d0
d1
d4
*
C
MFI Tax (in CD4+)
d0
d1
d4
DMSO
Panobinostat 50 nM
Romidepsin 5 nM
D
% Tax+ cells (in CD4+)
P1
P2
P3
P5
d0
d1
d4
DMSO
Panobinostat 50 nM
Romidepsin 5 nM
E
4-1BB mRNA [rcn]
HIAP-1 mRNA [rcn]
FOXP3 mRNA [rcn]
d0
d1
*
DMSO
Panobinostat 50 nM
Romidepsin 5 nM

Supplementary Figure 5. Assessment of marker and Tax expression via flow cytometry, and qRT-PCR in patient PBMCs. (A-E) $CD4^+$ T-cells isolated from five HTLV-1 infected patients were treated with Panobinostat (50 nM), Romidepsin (5 nM), or the solvent control DMSO on d0. The cultured cells received a second treatment on d2, equivalent to a quarter of the initial treatment of d0. Cells were analyzed via **(A-D)** flow cytometry and **(E)** qRT-PCR on d0, d1, and d4. **(A)** Gating strategy to assess Tax protein expression. Doublets were excluded in an FSC-H vs. FSC-A plot. In the next step, cells stained by the LIVE/DEAD Fixable Near-IR Dead Cell Stain Kit were excluded. Subsequently, the CD4 positive ($CD4^+$) cells were selected for further analysis. Tax expression was assessed in the $CD4^+$ subpopulation. **(B)** Mean expression of CD4 in the viable cell population ± SE of the five patients is displayed and was compared to the DMSO treatment using Student's t-test (*, $p < 0.05$). **(C)** Intracellular Tax expression was analyzed via flow cytometry, based on previous LIVE/DEAD staining (Near-IR Dead Cell Stain Kit), CD4, and Tax (Lt-4) labeling. The arithmetic mean fluorescence intensity (MFI) of the five patient samples ± SE were compared to DMSO using Student's t-test (all $p > 0.05$). **(D)** The individual frequency of intracellular Tax protein expression is plotted for patients (P) 1, 2, 3, and 5. **(E)** Mean relative copy numbers (rcn) of *4-1BB*, *HIAP-1*, and *FOXP3* normalized to the house-keeping gene *β-Actin*, of the five patients ± SE were compared to the DMSO-treated cells using Student's t-test (*, $p < 0.05$).
